# Factors affecting mental health and happiness in the elderly: A structural equation model by gender differences

**DOI:** 10.1002/brb3.2549

**Published:** 2022-03-29

**Authors:** Zohreh Mahmoodi, Mansoureh Yazdkhasti, Mahnoosh Rostami, Nooshin Ghavidel

**Affiliations:** ^1^ Social Determinants of Health Research Center Alborz University of Medical Sciences Karaj Iran; ^2^ Non‐communicable Diseases Research Center Alborz University of Medical Sciences Karaj Iran; ^3^ Dietary Supplements & Probiotic Research Center Alborz University of Medical Sciences Karaj Iran; ^4^ Health Systems Evaluation & Evidence Provincial Clinical Excellence Alberta Health Services Alberta Canada

**Keywords:** elderly, happiness, mental health, path analysis, psychological status

## Abstract

**Purpose:**

There are few studies on the gender differences in mental health, happiness, and their related factors among the older population through the structural equation model (SEM) in Iran. We conducted this study to evaluate the factors affecting mental health and happiness in the elderly using an SEM by gender differences.

**Methods:**

A cross‐sectional study was conducted on 739 elderly people in 2019 in Karaj, Iran. Sociodemographic, Symptom Checklist‐90‐Revised (SCL90‐R), and the Oxford Happiness Inventory were applied to evaluate the relationships between happiness, mental health, and sociodemographic factors by using statistical path analysis with Lisrel 8.8 and SPSS‐17.

**Results:**

Overall, 55.5% of the participants in the study were female. The SCL90 (*p* value = .000) and happiness (*p* value = .000) scores showed significant differences between men and women. Fit indices confirmed the high model fitness, desirability, and logical relationships between the variables according to the conceptual model in both men (*X*2 = 3.2, df = 1) and women (*X*2 = 5.4, df = 2) groups. According to the path analysis, among the variables that affected happiness just through the direct path, education had the most positive causal relationship in men (*B* = .13) and women (*B* = .16), but mental health problems in men (*B* = −.33) and women (*B* = −.26), as well as the distance from home to the healthcare center in men (*B* = −.13) and women (*B* = −.11), had the most negative causal relationship with happiness respectively. Age was the only variable that was negatively related to happiness through direct and indirect paths in the women (*B* = −.188).

**Conclusion:**

We provided an empirical model that illustrates the relationships between happiness, mental health, and related factors in the older population. Gender differences in path analysis showed that age negatively affects the happiness of older women but not men.

## Graphical abstract

We provided an empirical model that illustrates the relationships between happiness, mental health, and related factors in the older population. According to the path analysis, among the variables that affected happiness just through the direct path, education had the most positive causal relationship in men and women.

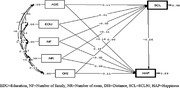

## 1 INTRODUCTION

The aging population is a global trend with extensive social and economic consequences. The World Health Organization (WHO) has predicted that the proportion of the world's elderly people will be doubled by 2050 from 12% to 22% (Organization, 2013). In 2050, 80% of all older people will live in low‐ and middle‐income countries, which are not ready to deal with the phenomenon of aging and social/economic consequences (Organization, 2018). Iran is facing the aging phenomenon due to the decrease in the birth rate in the past two decades, medical‐health advances, and increased life expectancy. The older population of Iran is expected to increase threefold in the next four decades (Afshar et al., 2016; Reza Amini & Sahaf, 2013).

Karaj city is the capital of Alborz province, and it is one of the most populous and immigrant cities in Iran, which is adjacent to the capital. The aging rate in this province has been increasing in recent years. The elderly are 10% of Iran's population, and over the next 30 years, more than 30% of Iran's population will be over 60 years old (Heidari, 2019).

Aging is associated with various challenges such as loneliness, the loss of a spouse, increased chronic diseases, lack of family/social support, and a lack of financial independence (Bakhtiyari et al., 2017; Ong et al., 2016; Tastan et al., 2019). The retirement and unemployment of the elderly have a very negative effect on their physical and mental health because, in these conditions, they are isolated and are away from their favorite job (Mandal & Roe, 2007; Minami et al., 2015).

Approximately 15% of the older population suffers from a mental disorder (Organization, 2013). Studies have shown increase of psychological symptoms with aging, including depression, anxiety, somatization, and obsessive‐compulsive disorders (Harandi et al., 2020; Hessel et al., 2001; Zis et al., 2017). Psychological problems reduce the feeling of happiness and threaten mental health (Brosschot et al., 2006). Happiness is a positive concept that is important for health and for maintaining it. Happiness is a kind of evaluation of a person's life. It includes life satisfaction, a positive mood, and the absence of depression/anxiety (Diener et al., 2018). It can increase our sense of meaning, abilities, and range of thinking.

Previous reports have provided evidence regarding the relationship between various socioeconomic variables such as age, education, income, occupation, gender, and race with psychological problems and happiness in the elderly (Barua et al., 2010; Murata et al., 2008; Tan et al., 2019). Availability of the healthcare system is another factor that can affect the health of the elderly. The primary source of mental health care for the elderly is their primary care providers in the field of physical health. (Gamm et al., 2010; German et al., 1985).

Significant differences in the mental health and happiness of the elderly have been shown in terms of gender (Hessel et al., 2001; Janus & Smrokowska‐Reichmann, 2019; Magni et al., 1985). A meta‐analysis has shown that older women reported significantly lower well‐being and less positive self‐concept than men (Pinquart & Sörensen, 2001).

Happiness and mental health are very important in the elderly because they can influence physical functioning and health (Veenhoven, 2008). Therefore, recognizing the factors involved can help us reduce the problems that societies will face in the future. To achieve this goal, the existing conditions, problems, and needs must be evaluated scientifically and professionally. There are few studies on the gender differences in mental health, happiness, and their related factors among the older population through path analysis in Iran. The novelty of our study was using the structural equation model (SEM) in men and women separately. Other studies have performed regression analyses that examine relationships between variables directly, but SEM in our analysis examines the relationship between variables through direct and indirect paths. Therefore, this study was conducted to investigate the factors affecting the mental health and happiness in the elderly using an SEM by gender differences.

## 2 MATERIALS AND METHODS

### 2.1 Design and participants

A cross‐sectional study was conducted on 739 elderly people in 2019 in Karaj, Iran by using multistage random sampling. We selected some healthcare centers at random in five different districts of Karaj city, and then the elderly who met the inclusion criteria were interviewed by trained experts. The inclusion criteria were people over 60 years of age, residing in the city of Karaj at the time of the study, with no history of psychiatric disease under treatment, ability to answer questions, and willingness to participate in the study. Exclusion criteria include the older population with a history of dementia, Alzheimer's disease, the mentally disabled, and terminal or chronic diseases, such as cancer and multiple sclerosis. The study protocol was approved by the Ethics Committee (IR.ABZUMS.REC.1396.13).

### 2.2 Measures

The questionnaire consisted of three sections: sociodemographic, Symptom Checklist‐90‐Revised (SCL90‐R) to measure mental health, and the Oxford Happiness Inventory (OHI) for happiness. The sociodemographic questionnaire consisted of 13 questions including age, gender, educational level, marital status, occupation, income, place of residence, the distance from home to the health center, the number of family members, the number of rooms, insurance, and the history of the disease.

The OHI (Argyle & Crossland, 1987) was introduced by Argyle and Crosland in 1987. It includes 29 items that are presented in four incremental levels, numbered from zero to three and the total score was noted from 0 to 87. The validity and reliability of OHI were evaluated by studies in Iran and other countries (Alipour & Noorbala, 1999; Hills & Argyle, 2002).

The SCL90‐R (Derogatis, 1977) is a 90‐item self‐report instrument that helps to evaluate a broad range of psychological problems and symptoms of psychopathology across nine subscales including (a) somatization, (b) obsessive‐compulsive, (c) interpersonal sensitivity, (d) depression, (e) anxiety, (f) hostility, (g) phobic anxiety, (h) paranoid ideation, and (i) psychoticism, which generally associated with mental health pathology and three global scales (Global Severity Index, Positive Symptom Distress Index, and Positive Symptom Total). Respondents were asked to rate the severity of their symptoms on a scale of 0–4 (0 = not at all, 1 = a little bit, 2 = moderate, 3 = quite a bit, or 4 = extreme). The instrument has been found to have a high construct validity as well as a high concurrent validity with similar instruments in Iran (Ardakani et al., 2016; Mirzaee, 1980).

The questionnaires were completed by trained experts through interviews conducted with subjects referring to the healthcare centers.

### 2.3 Research variables

Sociodemographic characteristics included age, gender, nationality, marital status (single, married, divorced, widow), educational level (years of education), occupation (unemployment, self‐employment, state‐employment, and retired), income, insurance, living area (slum/rural, urban), history of diseases, number of house rooms, the number of families, a distance of the home to healthcare centers, happiness, and psychological status. Variables used in the path analysis included age, education, distance from home to a healthcare center, number of house rooms, number of families, happiness, and mental health.

### 2.4 Statistical analysis

The demographic characteristics of the study population were summarized using descriptive statistics, mean (SD) for continuous variables, and frequency (%) for categorical variables. The mean (SD) of the SCL90 and the happiness scores were compared between men and women using a two‐tailed *t*‐test.

The Pearson correlation coefficient was calculated to evaluate the relationships among the variables. Then, the collected data were analyzed using path analysis. Path analysis was applied to evaluate the relationships among sociodemographic status, happiness, and mental health. Path analysis is a statistical method that can be used to analyze relationships between a set of independent variables and a dependent variable. Path analysis is an extension of the regression model, which researchers use to test the fit of a correlation matrix with a causal model that they tested (Wright, 1921).

Concerning the fitness indices of models in path analysis, chi‐square degrees of freedom index (*χ*2/def.) < 3 is preferred, even though some researchers consider a score of 4 and even 5 to indicate a good fit. Other indices for fitting the model include the normed fit index, comparative fit index, and the goodness of fit index, with preferred values >0.9. In the root mean square error of approximation (RMSEA) criteria, a score of ≤0.05 indicates a good fit and up to 0.08 is acceptable, although some sources consider a score up to 0.11 acceptable. We used SPSS‐17 and Lisrel‐8.8 software for data analysis with the application of path analysis.

**FIGURE 1:**
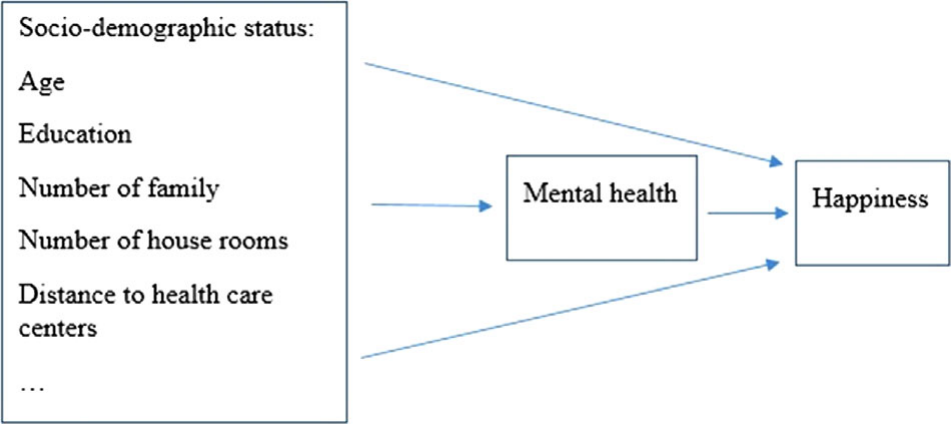
Hypothesized conceptual framework of the relationships among the study variables

A conceptual framework was investigated by using path analysis according to previous researches (Barua et al., 2010; German et al., 1985; Murata et al., 2008; Tan et al., 2019) (Figure 1).

## 3 RESULTS

Of 733 participants in the study, 407 (55.5%) were female and 326 (44.5%) were male. The mean age of the female and male respondents was respectively 66.095 ± 6.03 and 66.94 ± 5.9 years. The mean score of SCL90 and happiness of females were 77.16 ± 56.62 and 49.55 ± 18.50 and in the males were 61.15 ± 50.25 and 54.84 ± 18.66, respectively. Two‐tailed *t*‐test analysis showed significant differences in the SCL90 (*p* value < .001) and happiness (*p* value < .001) scores between men and women. The educational level was significantly higher in men than in women elderly (*p* value < .001). Demographic characteristics of participants, happiness score, SCL90‐R, and its subscales were shown in Table 1.

**TABLE 1:** Demographic characteristics of the study population

Variable		Mean ± SD	Variable		Mean ± SD
Age	Women	66.095 ± 6.03	Anxiety	Women	7.407 ± 6.58
	Men	66.94 ± 5.9		Men	5.1 ± 5.2
Education (years)	Women	3.49 ± 3.44	Obsessive‐compulsive	Women	10.1 ± 7.5
	Men	5.7 ± 4.6		Men	8.39 ± 6.6
Distance of healthcare centers (min)	Women	14.09 ± 8.9	Anger–hostility	Women	3.87 ± 3.7
	Men	14.78 ± 9.04		Men	4.5 ± 4.6
Number of family	Women	2.64 ± 1.46	Interpersonal sensibility	Women	8.8 ± 6.7
	Men	3.22 ± 1.5		Men	7.06 ± 6.1
Number of house rooms	Women	1.55 ± .7	Somatization	Women	12.45 ± 9.2
	Men	1.7 ± .69		Men	8.9 ± 8.04
Happiness	Women	49.55 ± 18.50	Psychoticism	Women	5.5 ± 6.02
	Men	54.84 ± 18.66		Men	4.6 ± 5.32
Total SCL90‐R	Women	77.16 ± 56.62	Paranoid ideation	Women	5.67 ± 4.4
	Men	61.15±50.25		Men	5.3 ± 4.4
Depression	Women	12.34 ± 9.7	Phobic anxiety	Women	4.4 ± 5.2
	Men	8.6 ± 8.4		Men	2.9 ± 4.2

Bivariate analysis in the females showed that mental symptoms on the SCL90 and education had respectively the highest negative (*r* = −.294) and highest positive (*r* = .205) correlation with happiness. In the men, mental symptoms on the SCL90 and education had respectively highest negative (*r* = −.332) and highest positive (*r* = .125) correlation with happiness (Table 2).

**TABLE 2:** Correlation between psychological status, happiness, and sociodemographic variables (male = 325 and female = 407)

Variable		AGE	EDU	NF	NR	DIS	SCL	HAP
AGE	Women	1	−0.215 **	−0.143 **	−.006	.167 **	.118 *	−.241 **
	Men	1	−0.170 **	−0.116 *	000	−.045	−.034	.081
EDU	Women		1	0.114 *	.244 **	.027	−.046	.205 **
	Men		1	.074	.210 **	.014	−.047	.125 *
NF	Women			1	.203 **	.05	−.12 *	.067
	Men			1	.131 *	.058	−.046	−.064
NR	Women				1	.006	−.126 *	.098 *
	Men				1	.064	−.67	.048
DIS	Women					1	.026	−.137 **
	Men					1	−.002	−.133 *
SCL	Women						1	−.294 **
	Men						1	−.332 **
HAP	Women							1
	Men							

** Significant at the 0.01 level (two‐tailed).

* Significant at the 0.05 level (two‐tailed).

EDU, education; NF, number of family; NR, number of room; DIS, distance; SCL, SCL90; HAP, happiness.

In the present model, the validity and reliability of the SCL90‐R instrument were evaluated in the model. Based on the results of composite reliability (CR), the average variance extracted (AVE), the maximum shared variance (MSV), and the average shared variance (ASV) were calculated to determine the convergent and divergent validity. Results showed CR > AVE and AVE > 0.5; therefore, there is convergent validity between the domains of these tools, as well as given that MSV < AVE and ASV < AVE, divergent validity is also desirable (Table 3).

**TABLE 3:** The results of confirmatory factor analysis, composite reliability (CR), and average variance extracted (AVE)

	Alpha Cronbach	Composite reliability	Average variance extracted	Rho‐A	MSV	ASV
Scl91	.957	.964	.747	.959	0.551	0.439

**FIGURE 2:**
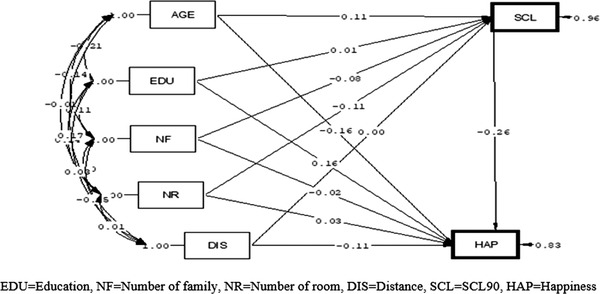
Path analysis diagrams of association of demographic factor and SCL90 with happiness in men

According to the path analysis in men, among the variables that affected happiness just through the direct path, the variable of education had the most positive causal relationship (*B* = .13), but mental health problems (*B* = −.33) and distance from home to healthcare center (*B* = −.13) had the most negative causal relationship with happiness. In other words, with the increase of one year to the educational years, the rate of happiness increased, and with the increase of one SCL90 score, the rate of happiness decreased. Other paths were not significant in the men (Figure 2).

**FIGURE 3:**
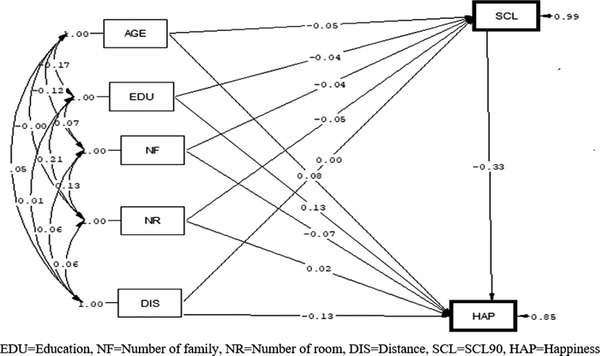
Path analysis diagrams of association of demographic factor and SCL90 with happiness in women

According to the path analysis in the women, among the variables that affected happiness just through the direct path, the variable of education had the most positive causal relationship (*B* = .16), but mental health problems (*B* = −.26) and distance from home to healthcare center (*B* = −.11) had the most negative causal relationship with happiness. Age was the only variable that was negatively related to happiness through both direct and indirect paths so that, with the increase of 1 year of age in women, the happiness score decreased (*B* = −.188). Mental health mediates the relationship between age and happiness in the indirect path (Figure 3 and Table 4).

**TABLE 4:** Direct and indirect effects of psychological status and sociodemographic variables on happiness

Variables		Direct effect	In direct effect	Total effect	*T*‐value	*R*2
Men	AGE	.08	.0165	.0965	1.45	.15
	Education	.13*	.00013	.13*	2.36	
	Number of family	−.07	.0132	−.056	1.41	
	Number of room	.02	.0165	.036	.33	
	Distance of care service	−.13*	−.0003	−.13*	2.50	
	SCL90	−.33*	−	−.33*	6.31	
Women	AGE	−.16*	−.029*	−.188*	3.34	.17*
	Education	.16*	−.0026	.16*	3.22	
	Number of family	−.02	.0208	.0008	.35	
	Number of room	.03	.0286	.0586	.62	
	Distance of care service	−.11*	−.0002	−.11*	2.34	
	SCL90	−.26^∗^	−	−.26*	2.34	

* Significant.

Fit indices confirmed the high model fitness, desirability, and logical relationships between the variables according to the conceptual model in both men (*X*2 = 3.2, df = 1) and women (*X*2 = 5.4, df = 2) groups (Table 5).

**TABLE 5:** Characteristics of the goodness of fit of path analysis model

Model	*X*2	df	CFI	GFI	NFI	RMSEA
Women	5.4	2	.81	.85	.80	.01
Men	3.2	1	.79	.86	.81	.00

df, degree of freedom; CFI, comparative fit index; GFI, goodness of fit index; RMSEA, root mean square error of approximation.

## 4 DISCUSSION

Elderly people are under a lot of stress due to retirement, lack of financial independence, insufficient income, and the loss of friends and relatives (Nourshahi et al., 2011). In the present study, the symptoms of mental disorders were higher in women than in men. These findings extend to those of Ehsanmanesh (2001), confirming a higher prevalence of mental disorders in women than men in urban areas. It seems that women are at greater risk for mental disorders in old age. Previous studies have shown the effect of gender on mental disorders, well‐being, and happiness (Morell, 2006).

According to the path analysis, there was a negative relationship between mental health problems and happiness in elderly men and women. Also, mental health and other sociodemographic variables could predict happiness (*R*2 = .17) in elderly women. On the other hand, in older men and women, a positive relationship was seen between education and happiness. So that with increasing the educational years, the level of happiness increased.

Socioeconomic status (SES) is a structure that has several variables, such as educational level, occupation, income, wealth, and deprivation (Miner et al., 2014). Education is known as the center of gravity and the focal point of SES. In other words, education is the main indicator of SES, which plays a pivotal role in SES gradient analyses (Cutler et al., 2008). Education not only induces job opportunities and future income potential in life cycles but also creates more life skills. This enables them to access more information to promote health (Lantz et al., 1998; Morell, 2006). Kong et al. (2019) found that low education in the elderly was associated with low income and other factors such as mental health, food insecurity, unqualified housing, and lack of insurance coverage. They found illiteracy and low education were more common in older women than men. The results of a study showed that education had a positive effect on general health of older population (Harandi et al., 2020).

In our study, education was evaluated as the most basic variable of SES in older men and women. We found that the average education in women was lower than in men. Studies have shown that higher levels of education lead to appropriate strategies for physical and mental health in the elderly; therefore, the elderly with higher education acquire higher health literacy that can improve mental health and happiness in them (Kong et al., 2019; Yang et al., 2020).

Distance from home to a healthcare center was another variable in our study, which was negatively related to happiness in both males and females through the direct path so that with increasing distance to a healthcare center, the happiness decreased. Studies have shown that increasing the distance to healthcare services causes increased mental disorder and decreased happiness in this group (Taheri Tanjanai et al., 2017; Záliš et al., 2016). The lack of proper access to healthcare had a negative impact on health and well‐being (Gu et al., 2009).

In the present study, age was the only variable that was associated with happiness only in women, both through the direct and indirect path. Aging declines the physical health of the elderly and decreases their quality of physical life. Physical and mental health have a reciprocal effect on each other so that reducing physical health can cause mental disorders in the elderly (Merghati‐Khoei et al., 2016).

In our study, mental health problems were negatively related to happiness through the direct path. Happiness is intertwined with mental health so that happiness protects old populations against stress and makes them more able to cope with their problems (Attari et al., 2018). Several variables affect the happiness of the elderly including gradual disability, reducing physical and mental strength, and decreased network of social interactions, which leads to their isolation. This issue can play a very important role in reducing the happiness and psychological well‐being of the elderly (Monirpour et al., 2019).

The growth of the aging population in Iran is one of the major challenges that the country will face in the coming decades. Therefore, planning to create welfare facilities and providing services to the elderly is one of the most important programs in Iran. Promoting the cultural level of the society, empowering the elderly, maintaining and promoting the physical, mental, social, and spiritual health of the elderly, strengthening social support to promote social capital, developing the necessary infrastructure in old age, and providing sustainable funding for the elderly care system are the goals of the National Document for the Elderly in Iran (Allameh, 2021).

One of the positive points of the present study is the evaluation of the validity and reliability of the SCL90 tool in the studied model. The convergent and divergent validity was desirable based on the results of the domains of the tool.

One of the limitations of our research was the low value of the coefficient of variance or *R*2 (*R*2 = .17), which is weak based on the three values expressed by Chin (1998) so that mental disorders and demographic variables could poorly predict happiness. Further studies with other variables need to be performed in the future.

## 5 CONCLUSION

We provided an empirical model that illustrates the relationships between happiness, mental health, and related factors in the older population. Mental health and other sociodemographic variables, including education and distance from home to healthcare centers, could predict happiness in the elderly. The most important factor that affects elderly happiness was mental health problems, which had a negative causal relationship with happiness. Our results revealed that older women had a lower educational level, higher symptoms of mental disorder, and lower happiness than men. The happiness and mental health of the elderly is one of the challenges in future societies. Iran will soon face an aging population, so it is necessary to evaluate the existing conditions and problems of the elderly, especially in the women's group in order to promote their happiness and physical and mental health by planning and adopting appropriate health policies in the elderly group.

## FUNDING

This study has been funded by Alborz University of Medical Sciences, Karaj, Iran.

## CONFLICT OF INTEREST

The authors declared no conflict of interest.

## CONSENT FOR PUBLICATION

All subjects participated voluntarily and signed an informed consent form.

### PEER REVIEW

The peer review history for this article is available at https://publons.com/publon/10.1002/brb3.2549

## Data Availability

Data is available on request from the authors.
